# Usefulness of Nonenhanced Computed Tomography for Diagnosing Urolithiasis without Pyuria in the Emergency Department

**DOI:** 10.1155/2015/810971

**Published:** 2015-09-01

**Authors:** Dong Hoon Lee, In Ho Chang, Jin Wook Kim, Byung Hoon Chi, Sung Bin Park

**Affiliations:** ^1^Department of Emergency Medicine, College of Medicine, Chung-Ang University Hospital, 102 Heukseok-ro, Dongjak-gu, Seoul 156-755, Republic of Korea; ^2^Department of Urology, College of Medicine, Chung-Ang University Hospital, 102 Heukseok-ro, Dongjak-gu, Seoul 156-755, Republic of Korea; ^3^Department of Radiology, College of Medicine, Chung-Ang University Hospital, 102 Heukseok-ro, Dongjak-gu, Seoul 156-755, Republic of Korea

## Abstract

We compared the clinical utility of nonenhanced computed tomography (NECT) and intravenous urography (IVU) in patients with classic symptoms of renal colic without evidence of a urine infection. This was a retrospective analysis of IVU and NECT performed in adult patients with suspected renal colic at an emergency department between January 2005 and December 2013. The records of all patients in NECT and IVU groups were reviewed, and the patients were categorized according to the cause of their symptoms. A total of 2218 patients were enrolled. Of these patients, 1525 (68.8%) underwent IVU and 693 (31.2%) underwent NECT. The patients in NECT group were older (45.48 ± 14.96 versus 42.37 ± 13.68 years,* p* < 0.001), had less gross hematuria (7.6 versus 2.9%,* p* < 0.001), and were admitted more often (18.6 versus 12.0%,* p* < 0.001) than the patients in IVU group. Urinary stones were detected in 1413 (63.7%) patients. NECT had a higher detection rate of urolithiasis than IVP (74.0 versus 59.0%,* p* < 0.001). No significant difference was observed in the incidence of urinary stones greater than 4mm between groups from the radiologic findings (*p* = 0.79) or the full medical record review (*p* = 0.87).

## 1. Introduction

Many patients visit the emergency department (ED) with renal colic caused by urolithiasis because it can cause severe and unbearable pain. The classic presentation of urolithiasis is renal colic characterized by colicky flank pain that radiates to the groin. Gross or microscopic hematuria is often seen in patients with urolithiasis; however, the absence of hematuria does not rule out a diagnosis of urolithiasis [1]. Management of urolithiasis in the ED includes pain control and diagnostic evaluation of the pain because urolithiasis is usually not severe. However, patients can suffer from severe pain, particularly during the first attack; thus, a definitive diagnosis of the cause of flank pain is necessary. Nonenhanced computed tomography (NECT) is a very useful diagnostic tool for evaluating urolithiasis in the ED and has been described as the best imaging tool for confirming the diagnosis of urolithiasis [2, 3]. And NECT is effective for detecting conditions other than urinary stones that can cause renal colic; it is less time consuming than intravenous urography (IVU), particularly in patients with obstructing calculi; it reduces the risk of complications from intravenous contrast media [4].

However, NECT is costly and exposes the patient to a large dose of ionizing radiation compared to IVU. Moreover, NECT does not influence patient-centered outcomes, such as the rates of diagnosis or hospital admissions, in patients with suspected urolithiasis [5–7]. Many patients with flank pain would not benefit from a NECT scan because most episodes of urolithiasis pass spontaneously. The choice of imaging modality should be based on accuracy, safety, cost-effectiveness, availability, and ease of interpretation [8, 9]. The present study compared the clinical utility of NECT and IVU in patients with classic symptoms of renal colic (flank pain, back pain, or both) without evidence of a urine infection and determined the clinical importance of NECT.

## 2. Materials and Methods

### 2.1. Study Design and Population

This study was a retrospective medical record review of patients that underwent IVU or NECT for suspected renal colic. It was approved by the Chung-Ang University Institutional Review Board (Seoul, South Korea). The study population consisted of adult patients who had visited the ED of the Chung-Ang University Hospital between January 2005 and December 2013. The inclusion criterion was undergoing IVU or a NECT scan during the study period. The exclusion criteria were as follows: IVU or NECT scan performed outside the ED, patients < 18 years of age, unavailable IVU and NECT scans, and reports dictated into a picture archiving communication system (PACS; Marosis, Marotech, Seoul, South Korea).

### 2.2. Nonenhanced Computed Tomography Protocol

All of the NECT studies were performed with a 256-MDCT scanner (Brilliance iCT, Philips Healthcare, Cleveland, OH, USA). All of the patients underwent a scan using the regular dose (RD) protocol from the proximal aspect of the T12 vertebra to the distal aspect of the symphysis pubis in the supine position. The RD protocol was acquired at a manually set peak tube voltage of 120 kVp with automated *z*-axis dose modulation by the scout image (DoseRight, Philips Healthcare, Cleveland, OH, USA). According to the RD protocol, the tube current was limited to 150 mAs. The remaining scanning parameters were as follows: detector configuration, 128 × 0.625; pitch, 0.915; beam collimation, 80 mm; rotation time, 0.4 sec; and helical acquisition.

Patients who underwent NECT with a low-dose protocol during the study period were not included in this study. The institution where this study was conducted had three different CT machines; however, only one CT scanner was used because the study participants included patients who had visited the ED, and the ED patients underwent NECT only on the CT scanner that belonged to the ED.

### 2.3. Categorization of the IVU and NECT Findings

Each of the IVU and NECT scans was classified into the following three categories based on a dictated report and an image review by a radiologist (Sung Bin Park) and three urologists (In Ho Chang, Jin Wook Kim, and Byung Hoon Chi): no cause of pain, urolithiasis, or a nonurolithiasis cause of pain. Urolithiasis was diagnosed if a stone was seen either in the kidney or in the ureter up to the distal ureterovesical junction (i.e., renal stones and proximal, mid, or distal ureteral stones, resp.). Stones were further subcategorized as large (>4 mm) or small (≤4 mm). If the image revealed a bladder stone and/or the dictated report specifically mentioned a passed stone, the case was categorized as urolithiasis. Asymptomatic renal stones were noted but not considered to be a cause of the symptoms. Based on a previous study, nonurolithiasis causes of pain were further categorized as follows: acutely important, follow-up recommended, and other unimportant causes [7]. Categorization, including the determination of the presence of flank/back pain and pyuria, was blinded to the separate record review.

### 2.4. Full Record Review

A full chart review was performed for each patient. Pyuria was considered present if more than 5 white blood cells/high power field were observed through microscopy. A full record review was conducted to categorize the findings, retrieve basic demographics, and document the presence of flank or back pain and pyuria. All of the physicians' and nurses' notes, the laboratory, imaging and pathology results, and the dictated operative reports and discharge summaries were reviewed in the electronic medical records, and the final diagnosis and management plan were abstracted.

### 2.5. Data Analysis

All of the parametric variables were evaluated using Student's *t*-test, and differences in nonparametric variables between the IVU and NECT groups were assessed using the chi-square test. All of the statistical analyses were performed using Excel (Microsoft Inc., Redmond, WA, USA) and SPSS (ver. 20.0 for Windows; Chicago, IL, USA), and a *p* value < 0.05 was considered to be statistically significant.

## 3. Results

**Figure 1:**
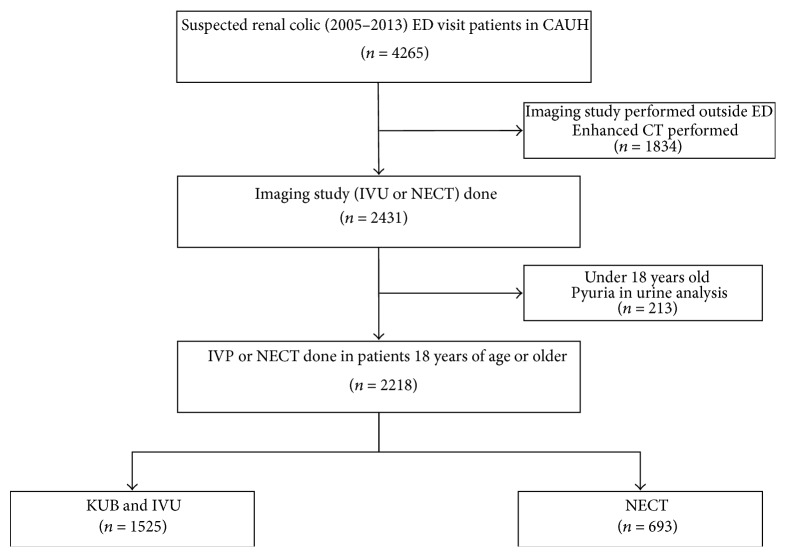
Flow chart of patient inclusion and review.

A total of 4,265 patients were diagnosed with renal colic in the ED during the study period, and 2,218 patients met the inclusion criteria. In total, 1,525 (68.8%) patients underwent IVU, and 693 patients underwent NECT (Figure 1). The patients' demographics, chief complaints, urine test results, and disposition are shown in Table 1. The patients in the NECT group were older (*p* < 0.01) and presented with hematuria less often (*p* < 0.05) than the patients in the IVU group; however, more NECT patients than IVU patients were admitted to the hospital (*p* < 0.01). The patient sex ratio was not different between the groups.

Table 2 shows the urolithiasis characteristics that were detected on the IV urogram and the NECT scan; a total of 1,603 urinary stones were detected in 1,413 patients (63.7%). The number of patients whose urinary stones were detected on an imaging study was higher in the NECT group than in the IVU group (74% versus 59%, *p* < 0.001). The NECT group had a higher proportion of renal stones, mid ureteral stones, and multiple stones than the IVU group (20.9% versus 13.8%, *p* < 0.001; 5.5% versus 2.7%, *p* < 0.001; and 14.6% versus 6.5%, *p* < 0.001, resp.); however, the proportion of distal ureteral stones was lower in the NECT group than in the IVU group (39.2% versus 49.1%, *p* < 0.001). The mean urinary stone size was smaller (3.62 ± 3.23 mm versus 4.15 ± 2.36 mm, *p* < 0.001) and the stones were more radiolucent (25.7% versus 13.2%, *p* < 0.001) in the NECT group compared to the IVP group.

The analysis and categorization of the causes of renal colic based on IVU, NECT, and full chart review are shown in Table 3. According to the radiological findings, of the 2,218 patients, no cause of pain was identified in 655 patients (29.5%), urolithiasis was identified as the cause of pain in 1,413 patients (63.7%), and a nonurolithiasis cause was found in 150 patients (6.8%). Among the 150 patients (6.8%) with a nonurolithiasis cause, 39 causes (1.8%) were classified as acutely important, 75 causes (3.4%) were classified as follow-up recommended, and 36 causes (1.6%) were classified as an unimportant cause. According to the full record reviews, no cause of pain was found in 632 patients (28.5%), urolithiasis was identified as the cause of pain in 1,433 patients (64.6%), and a nonurolithiasis cause was detected in 153 patients (6.8%). The NECT group had a higher incidence of urolithiasis and nonurolithiasis causes of renal colic but a lower incidence of no cause of pain on the radiological findings and the full chart reviews than the IVU group. However, the incidence of urolithiasis stones > 4 mm did not differ between the NECT and IVU groups (20.5% versus 20.0%, *p* = 0.79 on the radiologic findings, and 20.2% versus 20.5%, *p* = 0.87 on the full chart review). Moreover, the incidence of acutely important nonurolithiasis causes was similar between the NECT and IVU groups in the full record review (*p* = 0.41); however, the NECT group had a higher incidence than the IVU group on the radiological findings (*p* < 0.001).

**Figure 2:**
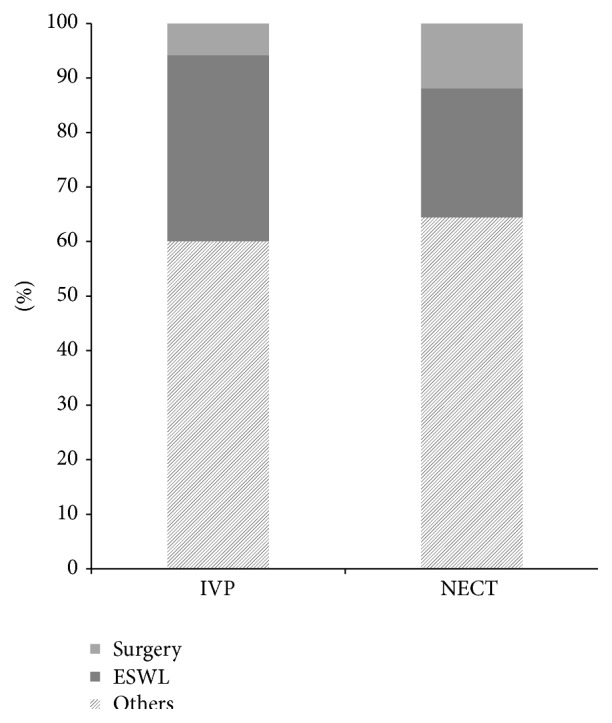
Urolithiasis treatment plan according to IVU or NECT. IVU: intravenous urogram; NECT: nonenhanced computed tomography; ESWL: extracorporeal shockwave lithotripsy; Others: medical expulsion therapy.

There was no difference in the urolithiasis treatment plan between the NECT and IVU groups (Figure 2). The incidence of active management, including surgery and extracorporeal shock wave therapy, did not differ between the NECT and IVU groups (35.4 versus 39.9%, *p* = 0.67), but the incidence of surgery was higher in the NECT group than in the IVU group (11.8 versus 5.8%, *p* < 0.001).

## 4. Discussion

**Figure 3:**
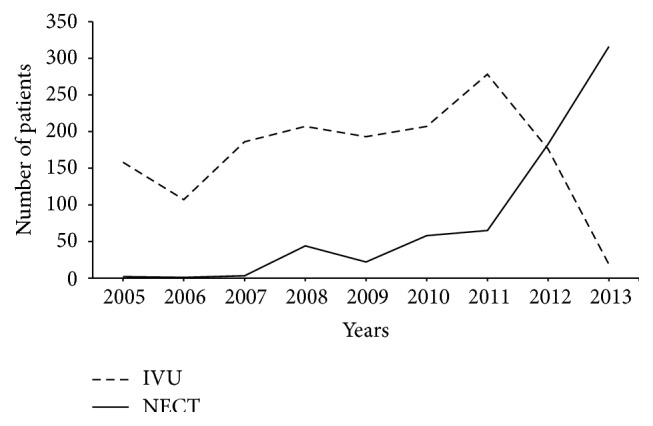
Changes in the use of imaging studies in patients with renal colic. IVU: intravenous urogram; NECT: nonenhanced computed tomography.

The prevalence of IVU and NECT use among renal colic patients admitted to the ED of our hospital is shown in Figure 3. The prevalence of NECT use was 1.25% in 2005 but increased to 94.9% in 2013. NECT has essentially replaced IVU in patients who attended the ED of our hospital with renal colic. A significant increase in the number of NECT scans performed on patients who were evaluated in the ED for renal colic was observed between 2005 and 2013, and more than a 10-fold increase was observed in the number of NECT scans that were performed in such cases. Larson et al. [10] reviewed CT use in a sample that included all patients who were evaluated in the ED and found a 16% compound annual growth rate, which outpaced the increase in CT use in in-patient and out-patient settings. Lim et al. [2] retrospectively reviewed the medical records of 2,180 patients with acute flank pain from 2008 to 2012 in an ED in Korea and reported that NECT use increased from 0.5% of the cases in 2008 to 66.6% of the cases in 2012. Reasons for this increase include easy accessibility, rapid image acquisition time, and superior image quality. In the current study, the ED physicians chose to use NECT for evaluating older patients and patients without microscopic hematuria (Table 1).

In the present study, NECT use resulted in a higher detection rate of renal stones and radiolucent stones than IVU use, and the size of the stones that were detected with NECT was smaller than those that were detected with IVU (Table 2). The incidence of urolithiasis stones > 4 mm did not differ between the two groups (Table 3). Several studies have reported that the sensitivity and specificity of NECT in diagnosing urolithiasis are 99% and 98%, respectively [11, 12]. However, higher accuracy may not always be necessary. Because urolithiasis is usually self-limiting, it can be managed conservatively [13]. Stones < 4 mm are likely to be passed spontaneously, and a consensus has not been reached on the necessity of determining the precise size and location of stones [14]. The complication rate from conservative management has been found to be as low as 7% when symptoms last < 4 weeks. Moreover, only 20% of patients with renal colic have stones > 4 mm [15]. Patients who have a very high probability of urolithiasis may not require high radiation imaging, such as NECT without a low-dose protocol, and can be managed with simple pain control and drugs that enhance the passing of the stone, with a definitive diagnosis made using a urine strainer.

In the present study, the full record review indicated that the incidence of acutely important nonurolithiasis causes was similar between the NECT and IVU groups; however, the radiologic findings indicated that the NECT group had a higher incidence of acutely important nonurolithiasis causes (Table 3). Moore et al. [7] reviewed 5,383 patients with renal colic who underwent NECT; 5.9% of these patients had an acutely important cause based on the radiological findings, but only 2.8% of the patients had an acutely important cause based on the full chart review. These results created two problems: the first was in determining how precise a NECT finding was for an acutely important cause of renal colic; the second was that although we routinely used NECT to detect renal colic, only 1.3% of the patients had an acutely important cause.

Three studies have evaluated alternative pathologies among patients with renal colic. Katz et al. [16] examined the NECT results of 1,000 patients with renal colic and found alternative or additional diagnoses in 101 of the patients. However, no comments were made regarding whether the findings required immediate or deferred treatment. Hoppe et al. [17] reported that 1,500 patients underwent a NECT scan due to renal colic and that 69% of the enrolled patients had urolithiasis. However, 14% of the patients had non-stone-related findings that required immediate or deferred treatment. Finally, Moore et al. [7] reported a nonurolithiasis diagnosis in 5.4% of cases, and, of those, 2.8% were categorized as acutely important. Each of these studies reported results that are similar to our result.

Among the urolithiasis treatment plans, the proportions of medical expulsion and observed cases did not differ between the NECT and IVU groups. NECT detected smaller stones that did not require management. Only 20% of the patients required admission for management, and 80% of the patients were ultimately discharged from the ED after simple pain control. Hyams et al. [5] reported that NECT use increased significantly between 2000 and 2008, but the proportion of patients who presented with renal colic and were ultimately diagnosed with nephrolithiasis remained stable at 20% during that time period. When the patients were diagnosed with a stone, approximately 85% were ultimately discharged from the ED and slightly more than 11% required hospital admission from the ED.

The usefulness of NECT for evaluating urolithiasis in the ED is undeniable, and NECT has been described as the best imaging tool for confirming the diagnosis of urolithiasis [2, 3]. NECT is effective for detecting conditions other than urinary stones that can cause renal colic; it is less time consuming than IVU, particularly in patients with obstructing calculi; it reduces the risk of complications from intravenous contrast media [4]. However, compared to IVP, there was no clinical advantage in using NECT for diagnosing urolithiasis. In addition, physicians should consider the high level of ionizing radiation exposure that results from NECT [18–20]. In this regard, some studies have focused on the use of “low-dose” NECT to reduce unnecessary ionizing radiation from imaging studies performed in patients with suspected renal colic. However, no large trials have been published on the use of low-dose NECT in diagnosing urolithiasis in the ED. We recently investigated the effects of low-dose NECT using iterative reconstructive (IR) algorithms in 69 patients with renal colic and compared its sensitivity with that of NECT [3]. IR reduces the image noise during image acquisition and can reduce the radiation dose from 5.77 to 1.34 mSV. The sensitivity and specificity of low-dose NECT remained at 96% and 100%, with positive and negative predictive values of 100% and 96.2%, respectively.

The present study was limited by the use of retrospective data and imaging reviews. Future studies should prospectively apply screening criteria and determine whether there are additional historical, physical, and point-of-care tests that may prevent or reduce the need for ionizing radiation in certain patients with suspected urolithiasis, while maintaining an acceptable risk threshold for not missing significant alternative diagnoses.

## 5. Conclusions

NECT is a rapid and accurate test for diagnosing suspected renal colic in the ED. However, when compared with IVP, it was not advantageous for detecting clinically significant urolithiasis or acutely significant causes of renal colic.

## Figures and Tables

**Table 1:** Patient demographics, chief complaints, urine test results, and disposition according to the full record reviews.

Characteristic	All	IVU	NECT	*p* value
	(*n* = 2218)	(*n* = 1525)	(*n* = 693)	
Age (years)	43.34 ± 14.16	42.37 ± 13.68	45.48 ± 14.96	<0.001
Sex (%)				0.130
Male	1574 (71.0)	1067 (70.0)	507 (73.2)	
Female	644 (29.0)	458 (30.0)	186 (26.8)	
Chief complaint (%)				
Flank pain or back pain	1415 (63.8)	869 (57.0)	546 (78.8)	<0.001
Abdominal pain	497 (22.4)	376 (24.7)	121 (17.5)	<0.001
Hematuria (gross)	136 (6.1)	116 (7.6)	20 (2.9)	<0.001
Others	170 (7.7)	164 (10.8)	6 (0.9)	<0.001
Hematuria in urine analysis (%)	1980 (89.3)	1376 (90.2)	604 (87.2)	0.032
Disposition				<0.001
Admission	312 (14.1)	183 (12.0)	129 (18.6)	
Discharge	1906 (85.9)	1342 (88.0)	564 (81.4)	

**Table 2:** Characteristics of urolithiasis detected by IVU and NECT.

Characteristic	All	IVU	NECT	*p* value
	(*n* = 2218)	(*n* = 1525)	(*n* = 693)	
Patients diagnosed with urolithiasis	1413 (63.7)	900 (59.0)	513 (74.0)	<0.001

Number of stones	1603	986	617	

Location of stone (%)				
Kidney	265 (16.0)	136 (13.8)	129 (20.9)	<0.001
Proximal ureter	523 (32.6)	320 (32.5)	203 (32.9)	0.90
Middle ureter	61 (3.8)	27 (2.7)	34 (5.5)	<0.001
Distal ureter	726 (45.3)	484 (49.1)	242 (39.2)	<0.001
Passed stone	28 (1.7)	19 (1.9)	9 (1.5)	0.48
Multiple stones (%)	154 (9.6)	64 (6.5)	95 (14.6)	<0.001
Radiopacity (%)				
Radiolucent (%)	284 (17.7)	128 (13.0)	156 (25.3)	<0.001
Size of stone (mm)	3.95 ± 2.74	4.15 ± 2.36	3.62 ± 3.23	<0.001

**Table 3:** Categorization of diagnoses in the IVU and NECT groups.

Characteristic	All	IVU	NECT	*p* value
	(*n* = 2218)	(*n* = 1525)	(*n* = 693)	
Radiologic finding (%)				
No cause of pain	655 (29.5)	586 (38.4)	69 (10.0)	<0.001
Urolithiasis	1413 (63.7)	905 (59.3)	508 (73.3)	<0.001
>4 mm	447 (20.1)	305 (20.0)	142 (20.5)	0.79
No urolithiasis cause				
Acutely important	39 (1.8)	14 (0.9)	25 (3.6)	<0.001
Follow-up recommended	75 (3.4)	18 (1.2)	56 (8.1)	<0.001
Other unimportant causes	36 (1.6)	1 (0.1)	35 (5.1)	<0.001
Full record review (%)				
No cause of pain	632 (28.5)	564 (37.0)	68 (9.8)	<0.001
Urolithiasis	1433 (64.6)	899 (59.0)	516 (74.4)	<0.001
>4 mm	450 (20.2)	308 (20.2)	142 (20.5)	0.87
No urolithiasis cause				
Acutely important	23 (1.0)	14 (0.9)	9 (1.3)	0.41
Follow-up recommended	94 (4.2)	29 (1.9)	65 (9.4)	<0.001
Other unimportant causes	36 (1.6)	1 (0.1)	35 (5.1)	<0.001
